# Comparison of Ischemic Stroke Outcomes in Patients With and Without Metabolic Syndrome: A Systematic Review

**DOI:** 10.7759/cureus.86464

**Published:** 2025-06-20

**Authors:** Jitender Sharma, Shrey Gondalia, Neetu Sharma

**Affiliations:** 1 Neurology, Army Base Hospital, New Delhi, IND; 2 Medicine, Jagruti Hospital, Jamnagar, IND; 3 Pathology, Military Hospital, Secunderabad, IND

**Keywords:** hypertension, ischaemic stroke, metabolic syndrome, mets, stroke

## Abstract

The present systematic review aims to compare ischemic stroke outcomes in patients with and without metabolic syndrome. The Preferred Reporting Items for Systematic Reviews and Meta-Analysis (PRISMA) guideline was followed during the conduct of this systematic review. The literature search encompassed extensive databases like PubMed, MEDLINE, ScienceDirect, and Cochrane Library. We included studies published between 2014 and 2024 in the English language that fulfilled inclusion and exclusion criteria. The quality of the included studies was assessed using appropriate tools tailored to the study design. The synthesis and analysis of data included a narrative summary of study characteristics, outcomes assessed, and main findings. The analyzed results demonstrate a multifaceted connection between metabolic syndrome and the outcomes of ischemic stroke. While several studies affirm that specific components of metabolic syndrome (MetS), notably hyperglycemia, hypertension, and waist circumference, are associated with increased risk and poor outcomes, the role of broader metabolic health appears nuanced. Some studies highlight that metabolic status (not solely weight or BMI) is paramount in predicting both acute events like early neurological deterioration (END) and recurrent strokes, suggesting tailored interventions focusing on metabolic health could enhance patient outcomes. This systematic review concluded that the risk of stroke and poor outcomes increased with the number of metabolic syndrome components like hypertension, elevated abdominal fat, and hyperglycemia.

## Introduction and background

Stroke is considered the second leading cause of death worldwide [1]. Ischemic stroke represents the predominant form of stroke, constituting around 62.4% of all stroke incidents globally in 2019 [2]. It occurs when the blood and oxygen supply to the brain is deficient, typically caused by blockages in blood vessels [3]. The global age-standardized incidence rate is projected to increase to 89.32 per 100,000 population by 2030 [2]. Ischemic strokes can be categorized into different types: large artery disease (about 20% of cases), caused by stenosis or occlusion of large cerebral arteries [4], lacunar and non-lacunar infarcts, and cardioembolic strokes [5].

Key risk factors include age (risk doubles over 55 years), hypertension, diabetes mellitus, dyslipidemia, smoking, physical inactivity, and cardiovascular diseases [2,3]. Metabolic syndrome represents a multifaceted group of metabolic disorders that markedly increase the likelihood of developing cardiovascular disease and type 2 diabetes [6-8]. It is characterized by a constellation of interconnected health conditions that share underlying pathophysiological mechanisms [9]. The group of metabolic disorders that characterize metabolic syndrome encompasses central obesity, insulin resistance, hypertension, and atherogenic dyslipidemia [10].

As defined by the World Health Organization (WHO) in 1999, metabolic syndrome (MetS) is characterized by the presence of insulin resistance or a fasting glucose level exceeding 6.1 mmol/L (110 mg/dl) or a 2-hour glucose level greater than 7.8 mmol/L (140 mg/dl). This condition must be present along with any two or more of the following criteria: HDL cholesterol levels below 0.9 mmol/L (35 mg/dl) in men and below 1.0 mmol/L (40 mg/dl) in women; triglyceride levels above 1.7 mmol/L (150 mg/dl); a waist/hip ratio greater than 0.9 for men or greater than 0.85 for women, or a BMI exceeding 30 kg/m2; and blood pressure readings greater than 140/90 mmHg.

The syndrome involves both genetic and acquired factors that contribute to inflammation and cardiovascular risk [8,11]. Metabolic syndrome is characterized by a cluster of interconnected clinical features that significantly increase cardiovascular and metabolic disease risks. Metabolic syndrome is a major risk factor for stroke, cardiovascular disease, and type 2 diabetes mellitus [12,13]. Metabolic syndrome (MetS) represents a critical cardiovascular risk factor with profound implications for ischemic stroke outcomes. Recent epidemiological research has illuminated the complex relationship between metabolic abnormalities and stroke progression. The prevalence of metabolic syndrome among ischemic stroke patients is substantial, ranging from 46.0% to 58.3% across diverse populations [14,15]. A comprehensive study demonstrated that MetS significantly increases the risk of poor outcomes in acute ischemic stroke patients, with an odds ratio of 2.48 (95% CI 1.29-4.78) [14].

Emerging evidence suggests a dose-response relationship between metabolic syndrome components and stroke risk. The risk of ischemic stroke rises steadily with the severity of metabolic syndrome (MetS). A hazard ratio of 1.75 (95% CI 1.35-2.27) for patients with high MetS severity [16]. Risk is particularly pronounced in specific demographic groups, notably white females (HR 2.63, 95% CI 1.70-4.07) [16]. Metabolic syndrome (MetS) significantly influences stroke recurrence risk through multiple mechanisms. Metabolic syndrome is associated with a 46% increased risk of stroke recurrence (relative risk 1.46, 95% CI 1.07-1.97) [17]. The likelihood of recurrence rises with the increasing number of components associated with metabolic syndrome [17]. The most significant predictors of stroke recurrence include low levels of high-density lipoprotein cholesterol (HDL-C) and the presence of ≥2 metabolic syndrome components [17]. Large waist circumference is associated with a 2.39-fold increased risk of stroke recurrence [18]. Central obesity may play a pivotal role in developing insulin resistance [18]. Obesity is associated with increased ischemic stroke incidence [19,20].

Patients with metabolic syndrome showed a higher recurrence rate (7.1% vs. 3.9% in patients without MetS) and a shorter time to stroke recurrence [18]. Although metabolic syndrome is clearly linked to the recurrence of strokes, the nature of this relationship is intricate. Some studies found the association becomes less significant when adjusting for individual metabolic components. Hypertension remains the single most important risk factor among metabolic syndrome components [21].

Metabolic syndrome increases stroke recurrence risk through multiple complex mechanisms. Metabolic syndrome triggers chronic inflammatory states that contribute to stroke recurrence through dysregulation of fat metabolism, inflammatory process proliferation in cardiovascular diseases, and endothelial dysfunction and vascular damage [22]. Vascular complications included impaired arterial repair mechanisms, increased coagulation risk, endothelial dysfunction, and potential insulin resistance-related vascular damage [17,22]. The continued increase in the incidence of MetS globally due to factors such as urbanization, dietary transition, sedentary lifestyles, and a growing incidence of obesity make it a significant public health issue. Such an increase among patients with high MetS rates is detrimental to populations already experiencing a heightened prevalence of cerebrovascular morbidity/mortality. This article seeks to fill this gap by performing a comprehensive analysis of ischemic stroke in patients with and without MetS. Hence, through in-hospital mortality, new neurological deficit, functional outcome, and recurrence rate, this study aims to determine the effect of MetS on stroke prognosis and comprehensively evaluate stroke outcomes in patients with and without metabolic syndrome.

## Review

Methodology

Search Strategy

The present systematic review used results from original publications and research papers that have been published on ischemic stroke and metabolic syndrome in databases including PubMed, MEDLINE, ScienceDirect, and Cochrane Library. The search terms employed a combination of MeSH terms and free-text keywords. Primary search terms included ‘ischemic stroke,’ ‘metabolic syndrome,’ and ‘MetS’ using the Boolean operators ‘AND’ and ‘OR.’

Studies considered for inclusion were published between 2014 and 2024. A preliminary screening was conducted by two independent researchers and resolved by a third reviewer if any disparities arose. A comprehensive examination of the title and abstract of each study confirmed their alignment with the research goals. Subsequently, the full text of the articles was obtained and meticulously examined to extract relevant outcome estimates, upholding a methodologically sound foundation for data collection.

Eligibility Criteria

Inclusion criteria include original articles, observational studies (cohort, case-control), clinical trials, randomized controlled trials (RCTs), retrospective studies, and studies published in English. Review articles, meta-analyses, case reports, case series, articles without full text, and studies written in other languages were excluded.

Data Extraction and Data Synthesis

Collected data were extracted and synthesized, creating a narrative summary encompassing study characteristics, outcomes, and findings. This systematic review followed the Preferred Reporting Items for Systematic Reviews and Meta-Analyses (PRISMA) guidelines, ensuring transparent and comprehensive reporting [23].

Result

**Figure 1:**
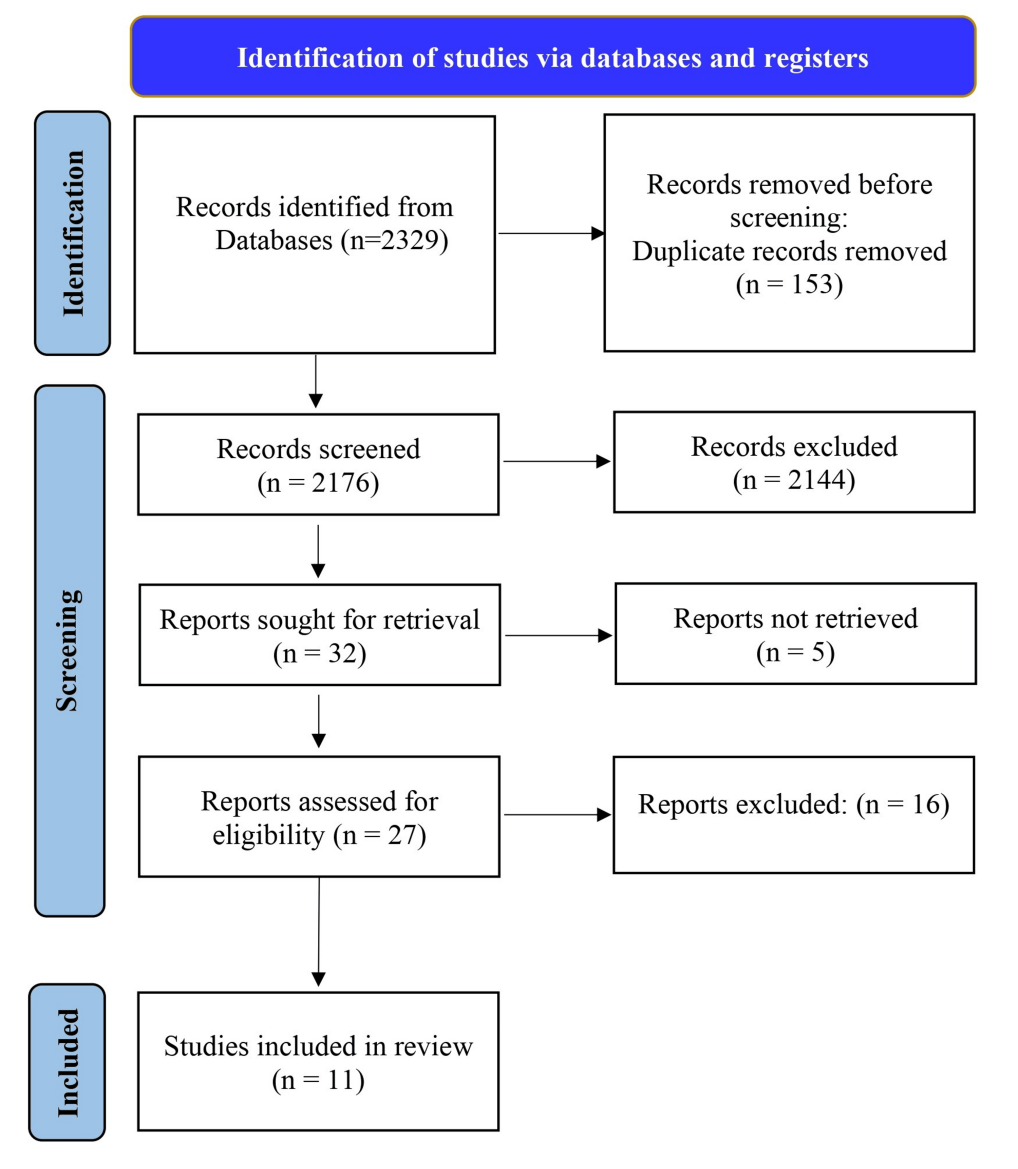
PRISMA flow diagram.

Figure 1 illustrates the studies included for analysis as per the PRISMA guidelines. A total of 2329 studies were initially searched from different databases using the search keywords. A total of 2176 records were screened after the initial exclusion of the studies. A total of 32 articles were selected for further consideration after a meticulous assessment of the titles and abstracts. Following that, five studies were eliminated based on the inclusion criteria. We screened 27 studies based on the inclusion and exclusion criteria. Finally, we selected 11 studies because of the non-availability of some data in the other studies.

Table 1 presents a comprehensive summary of various studies that investigate the relationship between metabolic syndrome (MetS) and ischemic stroke outcomes across different populations. The studies include diverse methodologies, such as cohort and cross-sectional designs, and cover several countries, including China, Korea, Poland, and Norway, with sample sizes ranging from 208 to over 35,105 participants.

**Table 1:** Characteristics of the included studies.

Author	Country	Design of the study	Sample size	Outcome assessed	Findings
Jia Q et al. 2014 [24]	China	Prospective cohort study	2639	Mortality, or stroke recurrence, modified Rankin Scale of 3 to 5 scores	Impaired glucose regulation (IGR) is an independent predictor for the mortality of patients with ischemic stroke.
Oh MY et al. 2014 [25]	Korea	Cross-sectional study	691	National Institutes of Health Stroke Scale (NIHSS) score, modified Rankin Scale (mRS) score	MetS may be a potent predictor of functional outcome after ischemic stroke.
Liu L et al. 2015 [15]	China	Retrospective study	530	National Institutes of Health Stroke Scale (NIHSS) score, stroke	The prevalence of MetS among patients with acute ischemic stroke in our study is 58.3%. Hyperglycemia is a significant predictor for poor functional outcomes.
Zhang X et al. 2016 [26]	China	Prospective cohort study	208	early neurological deterioration (END), Stroke	MetS may be a potential predictor for early neurological deterioration (END) after ischemic stroke.
Kang K et al. 2018 [27]	Korea	Retrospective study	1403	stroke severity, National Institutes of Health Stroke Scale (NIHSS) score	Abdominal obesity was associated with less baseline stroke severity in both men and women and associated with lacunar stroke in men.
Bembenek JP et al. 2018 [28]	Poland	Retrospective study	2048	modified Rankin Scale (mRS)	High waist-to-hip ratio (WHR) increased the odds of death or dependency at discharge. BMI shows the least clinical value in predicting stroke outcome in both genders.
Chen Z et al. 2020 [14]	China	Prospective study	248	modified Rankin scale score of 3-6, symptomatic intracranial hemorrhage (sICH), mortality	MetS is associated with poor prognosis in acute ischemic patients treated with endovascular thrombectomy (EVT).
Horn JW et al. 2021 [29]	Norway	Prospective cohort study	35105	Stroke	No increased risk for ischemic stroke for metabolically healthy overweight or obese. Ischemic stroke risk increased with the number of metabolic abnormalities, and the most important metabolic risk factor was hypertension.
Huang X et al. 2022 [30]	China	Cross-sectional study	856	Stroke	Metabolic abnormalities, rather than BMI, are significantly associated with recurrent stroke.
Ye J et al. 2023 [31]	China	Cross-sectional study	23,389	Stroke	A higher weight-adjusted waist index (WWI) is associated with a higher prevalence of stroke.
Yu XR et al. 2024 [32]	China	Retrospective study	456	National Institute of Health Stroke Scale (NIHSS), modified Rankin Scale (mRS)	TyG-WC and TyG-BMI were correlated with the severity and short-term outcome of new-onset acute ischemic stroke.

The primary aims of the studies were to assess various outcomes related to ischemic stroke, including mortality rates, functional outcomes, and neurological deterioration. For instance, Jia et al. [24] identified impaired glucose regulation as a significant risk factor for mortality but found no impact on dependency or stroke recurrence. In contrast, Oh et al. [25] concluded that MetS significantly predicted adverse functional outcomes, revealing a strong association with the number of MetS components.

Liu et al. [15] found a high prevalence of MetS among stroke patients yet asserted that MetS alone did not predict short-term prognosis, with hyperglycemia showing a detrimental effect on outcomes. Zhang et al. [26] highlighted a pronounced risk of early neurological deterioration associated with MetS, independent of inflammatory markers. Additionally, Kang et al. [27] reported that abdominal obesity was linked to lower baseline stroke severity, suggesting a complex interplay between obesity metrics and stroke manifestations. Bembenek et al. [28] provided insights into the predictive capabilities of waist-to-hip ratio versus waist circumference and BMI, revealing that high waist-to-hip ratios were predictive of worse outcomes, particularly in females. Furthermore, Chen et al. [14] and Huang et al. [30] both emphasized MetS's crucial role in predicting prognosis in stroke patients, finding strong associations between metabolic abnormalities and poor outcomes. Horn et al. [29] contributed to the discussion by examining metabolic health's relationship with ischemic stroke risk, concluding that metabolically healthy individuals with obesity do not face increased stroke risk, unlike those with metabolic abnormalities. Lastly, Ye et al. [31] and Yu et al. [32] expanded on the utility of specific indices such as the weight-adjusted waist index (WWI) and triglyceride-glucose index in predicting stroke prevalence and outcomes, respectively.

Overall, the collective findings illustrate a complex relationship between metabolic syndrome and ischemic stroke outcomes. While several studies affirm that specific components of MetS, notably hyperglycemia, hypertension, and waist circumference, are associated with increased risk and poor outcomes, the role of broader metabolic health appears nuanced. Some studies highlight that metabolic status (not solely weight or BMI) is paramount in predicting both acute events like END and recurrent strokes, suggesting tailored interventions focusing on metabolic health could enhance patient outcomes.

Critical Appraisal of Studies

We critically appraised our included articles using the Joanna Briggs Institute (JBI) checklist for cross-sectional studies [33] and the Risk of Bias in Non-randomized Studies - of Interventions (ROBINS-I) tool for prospective cohort studies and retrospective studies [34].

Table 2 presents a quality assessment of three included cross-sectional studies, focusing on key indicators of study integrity. We allocated a score of '1' to questions answered with 'yes' and a score of '0' to those answered with 'no,' 'unclear,' or 'not applicable (N/A).' Upon summing the total scores of all articles, we categorized the studies as low risk for scores between 6 and 8, moderate risk for scores between 3 and 5, and high risk for scores between 0 and 2. All three evaluated cross-sectional studies were found to be in the low-risk-of-bias category.

**Table 2:** JBI critical appraisal checklist for cross-sectional studies. JBI: Joanna Briggs Institute

JBI critical appraisal	Oh MY et al. 2014 [25]	Huang X et al. 2022 [30]	Ye J et al. 2023 [31]
Were the criteria for inclusion in the sample clearly defined?	1	1	1
Were the study subjects and the setting described in detail?	1	1	1
Was the exposure measured in a valid and reliable way?	1	1	1
Were objective, standard criteria used for measurement of the condition?	1	1	1
Were confounding factors identified?	1	1	1
Were strategies to deal with confounding factors stated?	1	1	1
Were the outcomes measured in a valid and reliable way?	1	1	1
Was appropriate statistical analysis used?	1	1	1
Total score	8/8 (100%)	8/8 (100%)	8/8 (100%)
Results	Included	Included	Included

**Figure 2:**
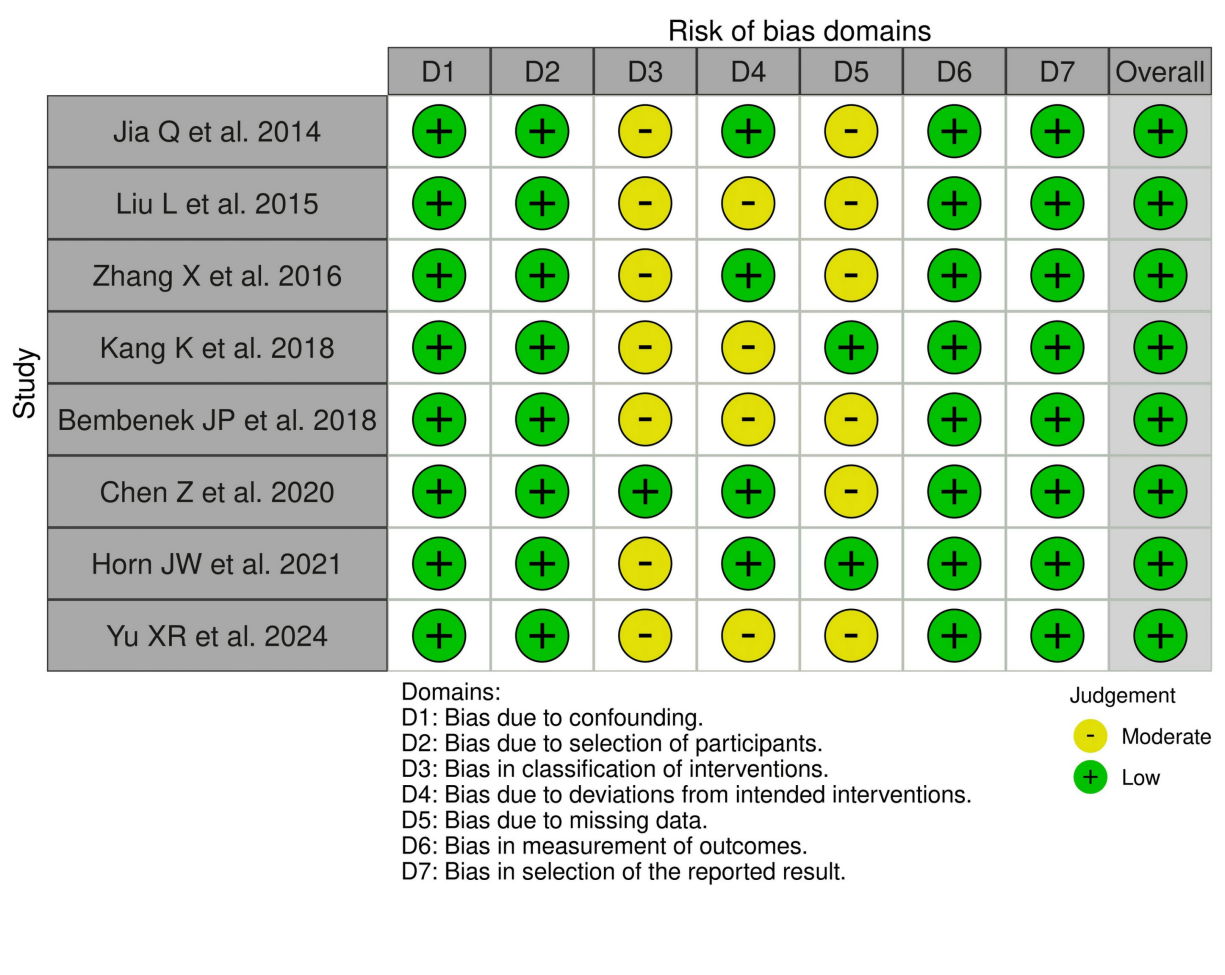
Traffic lights plot for the risk of bias for included prospective and retrospective studies. [14,15,24,26,27,28,29,32]

**Figure 3:**
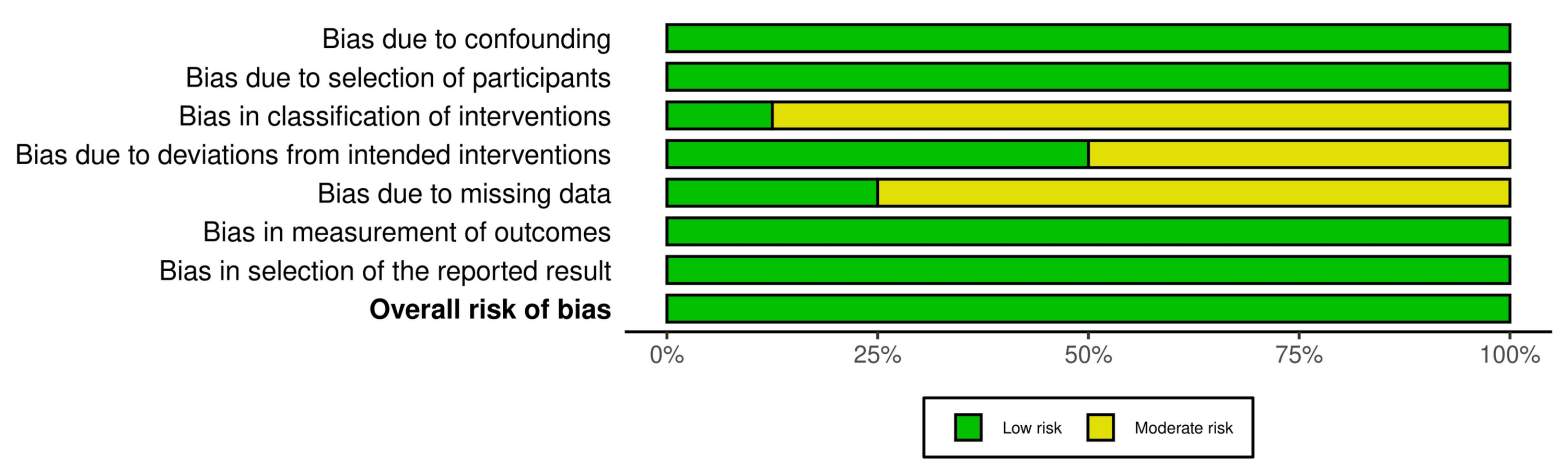
Summary of risk of bias assessment of included prospective and retrospective studies.

The risk of bias assessment result is demonstrated in Figures 2, 3. All seven domains of risk of bias of the ROBINS-I tool were thoroughly assessed for all the prospective and retrospective studies included in this systematic review. Overall, a low risk of bias was reported in all the included studies, as shown in Figures 2, 3.

Discussion

The present systematic review aims to compare ischemic stroke outcomes in patients with and without metabolic syndrome. Our study indicated that metabolic syndrome (MetS) is linked to a heightened risk of adverse outcomes in cases of ischemic stroke. The risk of poor outcomes increased with the number of metabolic syndrome components. Hyperglycemia was a significant predictor of poor functional outcomes.

The findings of our study align with several recent research publications that explore the relationship between metabolic syndrome (MetS) and ischemic stroke outcomes. Numerous studies have consistently shown that MetS is linked to heightened risks and a worse prognosis in patients suffering from ischemic stroke. Metabolic syndrome affects a significant proportion of ischemic stroke patients, with studies reporting prevalence ranging from 33.4% to 47.2% [14,35]. The risk of poor outcomes increases with the number of MetS components present [14,26]. Several studies support our findings of increased risks of ischemic stroke. A 2020 study found that patients with MetS had a significantly higher likelihood of poor outcomes at 90 days (odds ratio 2.48) [14]. A total of 63.2% of MetS patients had unfavorable functional outcomes compared to 44.0% without MetS. Overall mortality at three months was 19.0%. A separate study revealed that patients with MetS faced a 125% higher risk of experiencing early neurological deterioration (OR: 2.25) [26].

Research indicates that specific metabolic syndrome components contribute to stroke risks. High blood pressure, high triglycerides, and low HDL cholesterol were significantly associated with coronary heart disease in stroke patients [35]. Central obesity, in particular, was linked to stroke recurrence [18]. The increased risk may be attributed to insulin resistance, inflammatory processes, and cardiovascular complications associated with MetS components. Our study's findings align closely with several recent publications exploring the relationship between metabolic syndrome (MetS) and ischemic stroke outcomes. The key observations about increased risk and the role of hyperglycemia are supported by multiple research studies. Multiple studies corroborate our findings of increased risks associated with metabolic syndrome. A 2020 study of 248 ischemic stroke patients found that MetS was significantly correlated with poor outcomes, with an odds ratio of 2.48 (95% CI, 1.29-4.78) [14]. The risk of poor outcomes was positively associated with the number of MetS components, similar to our study's conclusion [14]. A 2015 study specifically noted that while MetS itself might not be predictive, hyperglycemia significantly impacted functional outcomes at 30 and 90 days [15].

Research consistently highlights the significant prevalence of MetS in stroke patients. The prevalence of MetS was reported to be 58.3% among patients with acute ischemic stroke [15]. Another study reported MetS in 46.0% of patients treated with endovascular thrombectomy [14]. While most studies show increased risks, some findings are more nuanced. Not all studies found significant correlations with mortality or symptomatic intracranial hemorrhage [14]. The risk varies across different demographic groups, with some studies noting higher risks in specific populations [16]. Researchers propose several mechanisms for MetS's impact, such as inflammatory state, activation of coagulation systems, increased cardiovascular disease risk, and potential genetic and lifestyle factors [36]. Metabolic syndrome (MetS) significantly impacts long-term stroke outcomes, with multiple studies revealing increased risks and poorer prognosis. Patients with metabolic syndrome demonstrate a 70% higher risk of incident stroke compared to those without MetS [37], an increased likelihood of stroke recurrence, and a higher risk of poor functional outcomes. Elimination of metabolic syndrome could potentially reduce overall stroke incidence by 19% and decrease ischemic stroke risk by 30% [38]. MetS patients also demonstrate higher resistance to thrombolysis and increase the likelihood of poor long-term neurological outcomes [39].

According to our research, abdominal obesity was linked to a lower baseline severity of stroke. Metabolically unhealthy participants had a higher ischemic stroke risk. Weight-adjusted waist index (WWI) positively correlated with stroke prevalence. The findings of the current study contribute to a growing body of evidence highlighting abdominal obesity's complex role in stroke risk, emphasizing the importance of metabolic health beyond traditional weight measurements. Multiple studies support the significant impact of abdominal obesity on stroke. Elevated waist circumference (WC) in women was significantly linked with increased stroke risk [40]. Abdominal obesity was identified as an independent and potent risk factor for ischemic stroke across different race-ethnic groups [41]. Research consistently demonstrates that abdominal obesity increases the risk for metabolic syndrome; metabolically unhealthy individuals show higher cardiovascular risk and waist circumference is a stronger predictor of stroke risk compared to body mass index (BMI) [42]. One longitudinal study showed a 12.4% stroke incidence in participants with abdominal obesity, compared to 6.8% in those without [43]. Each unit increase in waist circumference was associated with a 2-3% increase in stroke odds [43].

Our study's findings align with multiple research publications exploring the complex relationship between abdominal obesity and stroke risk. Abdominal obesity demonstrates significant associations with stroke risk, with notable variations across demographic groups. Some studies demonstrated that increased waist circumference strongly correlates with stroke prevalence [40,41,44]. In some studies, weight-adjusted waist index (WWI) positively predicts stroke risk [44,45]. Women exhibit a heightened risk of stroke associated with abdominal obesity [40,45]. In females, an increased waist circumference notably heightens the risk of stroke (OR 5.79, 95% CI 3.10-10.85) [45]. Waist-to-hip ratio demonstrates graded risk progression (second tertile OR 2.78, third tertile OR 7.69) [44]. Markers of abdominal fat are more precise indicators of stroke risk than body mass index (BMI) [41,44,46]. Waist circumference and related ratios demonstrate superior predictive value [41,44].

Future directions

Future research should focus on understanding metabolic syndrome's complex interactions with ischemic stroke, emphasizing personalized prevention and management strategies. The exact mechanisms linking MetS to stroke outcomes require further investigation. The predictive value of individual MetS components versus the syndrome as a whole needs more research.

## Conclusions

The present systematic review concluded that the risk of stroke and poor outcomes increased with the number of metabolic syndrome components like hypertension, elevated abdominal fat, and hyperglycemia. In our study, hypertension emerged as the most critical metabolic risk factor. Metabolic abnormalities were more predictive of stroke outcomes than Body Mass Index (BMI).

Our study's findings are consistent with a growing body of evidence suggesting that metabolic syndrome significantly impacts ischemic stroke outcomes, highlighting the importance of managing metabolic risk factors in stroke prevention and treatment. Proper management of metabolic syndrome components may significantly improve cardiovascular health and reduce stroke recurrence risk. Comprehensive, personalized management is crucial for improving ischemic stroke outcomes in patients with metabolic syndrome.
